# HIV community index testing reaches proportionally more males than facility-based testing and is cost-effective: A study from Gaza province, Mozambique

**DOI:** 10.1371/journal.pone.0286458

**Published:** 2023-05-26

**Authors:** Mário Songane, Célia C. Magaia, Aleny Couto, Nataniel Dengo, Abdul R. Cassamo, Rene Nhantumbo, Carlos Mahumane, Atanásio Mabote, Silvia Mikusova, Amâncio Nhangave, Nilesh Bhatt, Sushant S. Mukherjee

**Affiliations:** 1 Elizabeth Glaser Pediatric AIDS Foundation, Maputo, Mozambique; 2 Programa Nacional de Controle de HIV/SIDA, Maputo, Ministério da Saúde, Mozambique; 3 Direção Provincial de Saúde de Gaza, Xai-Xai, Ministério da Saúde, Mozambique; 4 Elizabeth Glaser Pediatric AIDS Foundation, Washington DC, United States of America; University of Zimbabwe Faculty of Medicine: University of Zimbabwe College of Health Sciences, ZIMBABWE

## Abstract

**Background:**

In Mozambique, 38.7% of women and 60.4% of men ages 15–59 years old living with HIV do not know their HIV status. A pilot home-based HIV counseling and testing program based on index cases in the community was implemented in eight districts in Gaza province (Mozambique). The pilot targeted the sexual partners, biological children under 14 years old living in the same household, and parents (for pediatric cases) of people living with HIV. The study aimed to estimate the cost-efficiency and effectiveness of community index testing and compare the HIV testing outputs with facility-based testing.

**Methods:**

Community index testing costs included the following categories: human resources, HIV rapid tests, travel and transportation for supervision and home visits, training, supplies and consumables, and review and coordination meetings. Costs were estimated from a health systems perspective using a micro-costing approach. All project costs were incurred between October 2017 and September 2018 and converted to U.S. dollars ($) using the prevailing exchange rate. We estimated the cost per individual tested, per new HIV diagnosis, and per infection averted.

**Results:**

A total of 91,411 individuals were tested for HIV through community index testing, of which 7,011 were newly diagnosed with HIV. Human resources (52%), purchase of HIV rapid tests (28%) and supplies (8%) were the major cost drivers. The cost per individual tested was $5.82, per new HIV diagnosis was $65.32, and per infection averted per year was $1,813. Furthermore, the community index testing approach proportionally tested more males (53%) than facility-based testing (27%).

**Conclusion:**

These data suggest that expansion of the community index case approach may be an effective and efficient strategy to increase the identification of previously undiagnosed HIV-positive individuals, particularly males.

## Introduction

Globally, an estimated 38.4 million people were living with HIV in 2021 of which only 28.7 million people had access to antiretroviral therapy (ART) and 5.9 million people were unaware of their HIV status [1]. While critical progress has been made in addressing the HIV epidemic, many countries are still not on track to reach the global UNAIDS 95-95-95 targets, which seek to ensure 95% of people living with HIV (PLHIV) know their HIV status, 95% of PLHIV receive ART and 95% of those on ART are virally suppressed by 2030.

HIV causes an estimated 38,000 deaths per year in Mozambique [2]. According to 2020 UNAIDS estimates, the prevalence of HIV in the population between ages 15–49 years old is 11.5%, 14.4% in women and 8.6% in men [2]. Of the 2.1 million PLHIV in Mozambique, only 1.4 million are receiving ART [2]. Identification of HIV-positive individuals would enable timely initiation of ART, leading to improved life expectancy and lower risks of opportunistic infections [3]. At the population level, the expansion of effective ART would reduce HIV transmission, consequently limiting the social and economic burden of the disease [3,4].

HIV testing and counseling are the first crucial steps for increasing rates of ART use and viral suppression. However, according to the latest UNAIDS estimates, 1.7 million of the 2.1 million estimated PLHIV know they are HIV-positive, meaning that 400 thousand (19%) people do not know they are HIV-positive in Mozambique [2]. A recent study by Lopez-Varela estimated that 75.9% of men and 88.9% of women were aware of their HIV status in Southern Mozambique [5]. Furthermore, the latest survey by Mozambique`s Ministry of Health (MoH) estimated that the coverage rate of HIV testing in people ages 15–49 years old was only 78% [6].

The government of Mozambique has implemented targeted strategies, including index testing, to improve the identification of PLHIV. Index testing focuses on offering HIV testing services to sexual partners, biological children under 14 years old living in the same household, and parents (in pediatric cases) of a known HIV-infected person. Index testing has been shown to be an efficient strategy to identify and enroll in ART previously undiagnosed individuals in various countries in sub-Saharan Africa, including in Mozambique [7–11].

Index testing can be done at the health facility (HF) or in the community. In Mozambique, facility-based index HIV testing is defined as the index-linked testing that take place within the health facility and is managed by the MoH, whereas community index testing is defined as those that take place in the community at the home of the index-linked individuals and is managed, due to MoH`s limited funds, by implementing partners such as the Elizabeth Glaser Pediatric AIDS Foundation (EGPAF). Many individuals are reluctant to go to HFs to do HIV testing for various reasons, with the primary reasons being concern about stigma, discrimination and cost of travelling [12,13]. To improve the yield of index testing, the government decided to implement community index testing through implementing partners. Under this strategy, healthcare workers include HIV testing and counseling in their other routine activities (i.e., health education, vaccination, etc.) in order to protect the privacy of those being tested for HIV.

Expansion of community index testing in Mozambique would accelerate the achievement of the first UNAIDS 95 goal, but expanding this approach requires assessing the resource implications of this scale-up. Information on index testing costs and efficiency in Mozambique is scarce. An in-depth cost analysis is needed to determine the affordability of this strategy and provide policymakers and planners with useful information to better inform how to plan and allocate resources for the expansion of index testing to the community level.

We investigated the costs, cost-efficiency and cost-effectiveness of community HIV index testing in eight districts in Gaza Province, Mozambique. Furthermore, we assessed how potential variations in inputs (such as the price of HIV rapid tests) could impact the cost-efficiency of index testing.

## Study design and methods

### Community index testing and study location

In October 2017, EGPAF, in collaboration with local community partners, implemented a pilot community HIV index testing program based on index cases identified in 102 HFs located in eight districts in Gaza province, namely: Bilene (9 HFs), Chibuto (16 HFs), Chókwè (24 HFs), Chongoene (13 HFs), Guijá (9 HFs), Limpopo (7 HFs), Manjakaze (16 HFs) and Xai-Xai (8 HFs). With an HIV prevalence of 24.4%, Gaza Province has the highest prevalence among all provinces in Mozambique [14].

The primary index cases were identified from those who tested positive during HF routine HIV testing, care and treatment services, and individuals who died of HIV. Primary index cases were extracted from the Open Medical Record System (Open MRS), a database containing routinely-collected demographic and clinical data of patients who receive HIV services. As part of the pilot, field officers (also known as community lay counselors) visited all identified HIV patients, adults and children, in their homes.

Each field officer received a list of index cases to visit in the community and performed counselling before HIV testing for all family members or sexual partners who agreed to be tested. The target populations were sexual partners of the index case, all biological children under 14 years old living in the same household of the index case, and, in pediatric cases (children under 14 years old), parents of the HIV-infected child. For each index case, a tracking form with information about contacts who belonged to these three high-risk groups was completed.

Index contacts who were known to be HIV-positive were not tested or included in the count for the community index case testing. Also, the primary index cases were not included in the count of community index cases. Furthermore, we did not include the costs of the primary index case test since the testing was done using HF resources and costs were incurred at the HF level. All clients diagnosed with HIV in the community were referred to the HF for care and treatment as per MoH guidelines.

### Index testing staff and activities

A total of 250 trained field officers conducted community index counseling and testing in the eight districts included in the pilot (October 2017 to September 2018). Supporting personnel involved in the pilot included one project coordinator, one deputy coordinator, one monitoring and evaluation (M&E) officer, eighteen supervisors, seven data entry clerks, one administrative assistant, one cashier, one office assistant, one driver and one accountant.

Based on information from the staff involved in the pilot and work agreements, we estimated that field officers spent 75% of their time on community HIV index testing and 25% of their time on tuberculosis (TB) screening and tracing of individuals who were lost to follow-up in the community. For the remaining staff, we sourced estimates of the percentage of their time dedicated to activities related to community index testing from their monthly activities report on Replicon, an online timesheet software used for human resources management. Human resources costs were then derived from these percentages and annual salaries.

As a routine part of the pilot, the project coordinator made five field visits per month to various districts for monitoring and supervision, and the deputy coordinator and M&E officer made ten field visits per month to monitor the use of registers and data collection instruments. Project management staff participated in quarterly coordination meetings, and there were two additional meetings per month held in the district capital attended by supervisors, data entry clerks, the M&E officer, the coordinator and deputy coordinator and field officers.

### Data collection and cost-efficiency analysis

Cost data on pilot-related activities in the eight districts were collected from internal financial reports and spreadsheets, databases, and relevant logbooks for a period between October 2017 and September 2018. Costs included human resources, HIV rapid tests, travel and transportation for home visits and supervision, supplies (i.e., stationary, smartphones for field officers, and personal protective equipment), training, and review and coordination meetings (S1A-S1D Table in S1 File). Costs were estimated from a health systems perspective using a micro-costing method, combining top-down and bottom-up approaches to obtain resource use and costs per line item.

Costs were aggregated across districts, because the financial system does not provide disaggregate district financial data. We focused on routine program implementation costs to understand how the community index testing program could be scaled up. All project costs were converted to U.S. dollars ($) using the prevailing exchange rate at the time of purchase or payment from Mozambique`s Central Bank. Since all costs were incurred in the same financial year, we did not adjust to 2018 US$. HIV testing services, in the context of community index testing, included the provision of both pre- and post-test counseling, first HIV testing, and confirmatory testing for a positive HIV result.

Trainings were treated as capital costs and annualized over two years, as previously done by Vyas *et al*. (2020) [15]. We applied a discount rate of 3% according to WHO guidelines [16] and annualized costs by dividing the total cost of the training by the annuity as described previously [17,18].

We recorded the total number of index cases tested for HIV and the number of index cases who were diagnosed with HIV through the community index testing approach. To obtain the cost per client tested for HIV and new HIV diagnoses, we calculated the total cost of community index testing in the reporting period and then divided it by the number of clients tested and the number of new HIV diagnoses, respectively. The cost estimation methodology was modelled based on a methodology described by Mwenge *et al*. [19] and Vyas *et al*. [15].

### Sensitivity analysis

A one-way sensitivity analysis was performed to assess the impact of variation of each input category on the cost per client tested for HIV and cost per new HIV diagnosis. The one-way sensitivity analysis consisted of varying each input category by applying a variation range of plus or minus 10% while the others remained the same as described previously [15] (S2 Table in S1 File).

### Cost-effectiveness

The seroincidence in individuals who are not in ART in sub-Saharan Africa is 13.0/100 person-years (PY) (0.13) whereas in individuals on ART is 8.5/100PY (0.085) [20].

Since clients who are diagnosed with HIV initiate ART immediately, we assumed that the HIV incidence drops from 0.13 to 0.085 after HIV diagnosis. Thus, we calculated the number of HIV infections averted per year by multiplying the number of new HIV diagnoses by the difference between HIV incidence before and after HIV diagnosis, and then multiplied by the year, as shown in the formula bellow as described previously [21]:

a=Nu*(Tu–Ta)*t

**where**: ***a*** is the number of HIV infections averted, ***Nu*** is the number of new HIV diagnoses, ***Tu*** is the incidence rate of individuals who are not on ART, ***Ta*** is the incidence rate of individuals who are on ART, and ***t*** is the year.

The cost per infection averted in each year was calculated by dividing the total cost of implementing the pilot community index testing by the number of infections averted. We estimated the cost per infection averted in year 1, 2 and 5.

### Facility-based HIV testing

Facility-based HIV testing includes all HIV tests done at HFs, such as provider-initiated counseling and testing (PITC), testing performed during medical male circumcisions, facility-based index testing and, voluntary counseling and testing (VCT) [22]. A study in a district in Southern Mozambique estimated that the vast majority of the individuals identified as new HIV diagnoses were from facility-based PITC (38%) and VCT (29%) [23].

The number of clients tested for HIV and the number of new HIV diagnoses between October 2017 and September 2020 in 102 HFs were extracted from Open Medical Records Systems from the eight districts included in the pilot. The reason the indicators data is from October 2017 to September 2020, unlike for cost data, which is from October 2017 to September 2018, was to assess the overall trend in testing and outcomes in the districts included in the study.

### Ethics statement

This evaluation was implemented under the auspices of EGPAF’s Patient and Program Outcomes Protocol (PPOP). Permission and ethical clearance to conduct this protocol was obtained from Mozambique`s Ministry of Health institutional review board (IRB) (approval number CNBS/656/19) and Advarra in the United States. PPOP is limited to the analysis of secondary data that are routinely collected as part of the standard services and does not pose added risks to the safety or rights of patients. This evaluation did not involve direct interaction with participants and informed consent was not required. No additional patient information was collected outside of the records at the time of data extraction.

## Results

### Costs and cost-efficiency

A total of 91,441 individuals were tested for HIV through community index testing, of which 7,011 (7.7%) were newly diagnosed with HIV. Human resources were the major cost driver (52%), followed by the purchase of HIV rapid tests (28%), supplies (8%), training (6%), communication and review meetings (3%), travel for supervision and home visits (3%) (Table 1 and S1A–S1D Table in S1 File). The cost per individual tested was $5.82, and the cost per new HIV diagnosis was $65.32 (Table 2).

**Table 1:** Total annual community index testing and counseling costs.

Category	Amount ($)	Percentage (%)
Human resources	299,245	52
Travel and transportation	14,215	3
Annualized training costs	34,820	6
Supplies	43,108	8
Communication and review meetings	18,680	3
Subtotal excluding HIV rapid test costs	410,068	72
Screening for HIV with Determine HIV rapid Test	122,522	21
Confirmation of HIV with Uni-Gold HIV rapid Test	38,522	7
Subtotal HIV rapid tests	161,044	28
**Total**	**571,112**	**100**

**Table 2:** Cost per client tested for HIV and cost per new HIV diagnosis.

Category		Value
Costs	Total annual costs excluding HIV rapid tests	$410,068
Number of clients	Clients tested	91,441
	New HIV diagnoses	7,011
Cost per client excluding the cost of rapid tests	Cost per client tested for HIV	$4.48
	Cost per new HIV diagnosis	$58.49
Price per HIV rapid test	Screening for HIV with Determine HIV rapid test	$1.34
	Confirmation of HIV diagnosis with Uni-Gold HIV rapid test	$5.49
Total cost per client	**Cost per client tested** *	**$5.82**
	**Cost per new HIV diagnosis** **	**$65.32**

*Includes only cost of Determine HIV rapid test.

**Includes cost of Determine and Uni-Gold HIV rapid tests.

There are no publicly available estimates of cost per client tested and per new HIV diagnosis in HFs in Mozambique. However, Mwenge *et*. *al*. 2017 estimated these costs (in 2016 $) for facility-based testing in Malawi, Zambia, and Zimbabwe [19]. These estimates were used for our analysis after adjusting to 2018 $ using U.S. consumer price index (S3 Table in S1 File). The estimated mean cost per client tested was $5.15 in Malawi, $4.44 in Zambia, and $9.20 in Zimbabwe. The mean cost per new HIV diagnosis was $83.26 in Malawi, $77.04 in Zambia, and $187.19 in Zimbabwe.

### Sensitivity analysis

**Fig 1:**
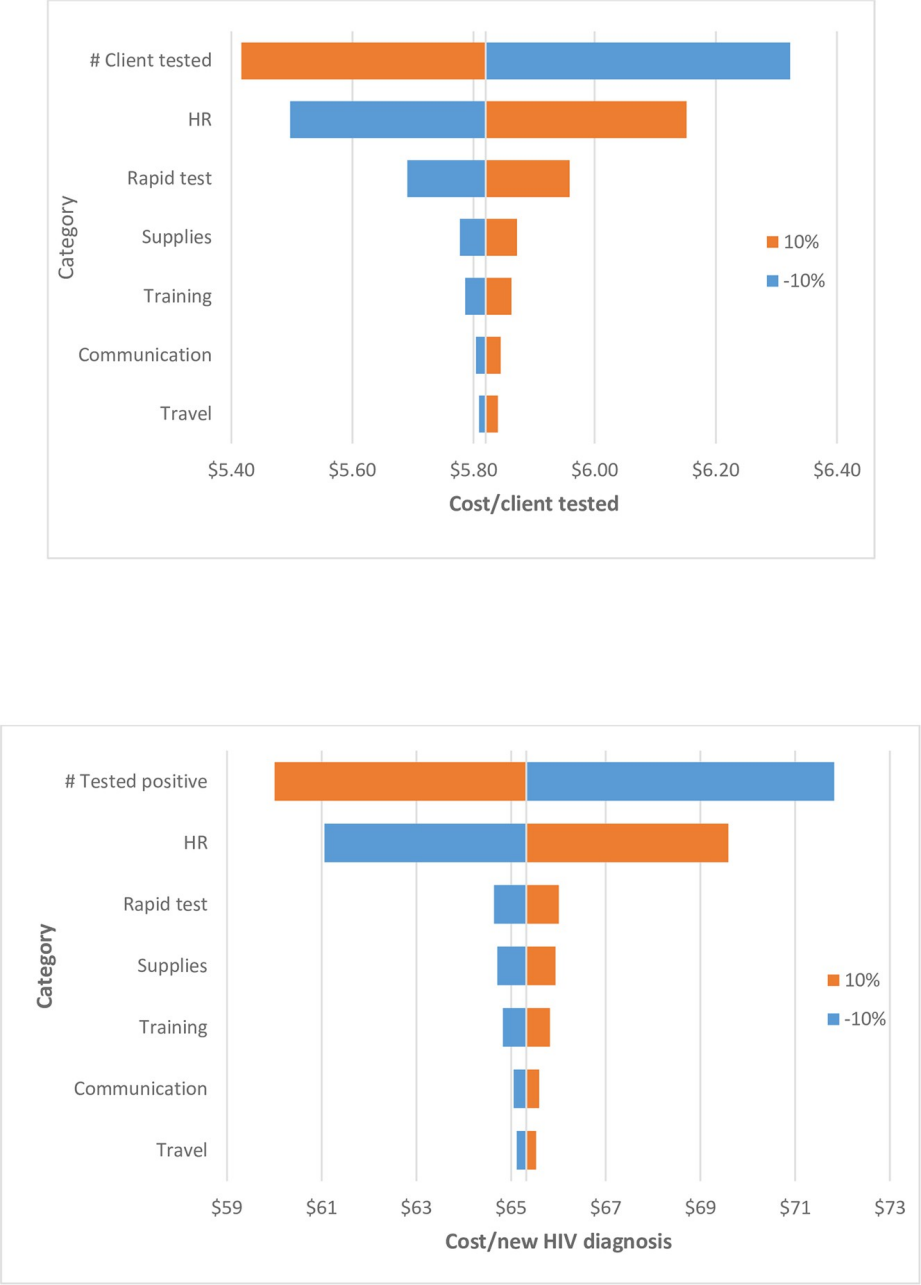
Sensitivity analysis. *Note*: Two valúes for each input category were used (+10%), the lowest in the range (blue) and highest in the t ange (orange), while the rest of the parameters remained the same. **a.** Tomado plot of one-way sensitivity analysis: Cost per client tested. *Note*: Two valúes for each input category were used (+10%), the lowest in the range (blue) and highest in the range (orange), while the rest of the parameters remained the same. **b.** Tornado plot of one-way sensitivity analysis: Cost per new HIV diagnosis.

When inputs were varied by plus (orange) or minus (blue) 10%, only human resources, number of clients tested (and new HIV diagnoses), and purchase of HIV rapid tests caused considerable variation in both cost per client tested and cost per new HIV diagnosis. The biggest impact was caused by varying plus or minus 10% in the number of clients tested (and new HIV diagnoses) which had an inverse correlation (Fig 1A and 1B and S2A and S2B Table in S1 File).

### Cost-effectiveness

We first calculated the number of infections averted (***a*)** in years 1, 2 and 5 through first multiplying the total number of new HIV diagnoses by the difference between incidence rate before (0.13) and after (0.085) HIV diagnosis, then multiplying by the year:

- Year 1: ***a =*** 7,011 * (0.13–0.085) * 1 = 315
- Year 2: ***a =*** 7,011 * (0.13–0.085) * 2 = 631
- Year 5: ***a =*** 7,011 * (0.13–0.085) * 5 = 1,577

Next, the total cost of community index testing was divided by the number of infections averted calculated above to calculate the cost per infection averted in each year:

- *Cost per infection averted in year 1* = $571,112/315 = $1,813
- *Cost per infection averted in year 2* = $571,112/631 = $905
- *Cost per infection averted in year 5* = $571,112/1,577 = $362

The estimated cost per HIV infection averted of the pilot community index testing in Gaza in years 1, 2 and 5 were $1,813, $905 and $362, respectively.

### Number of clients tested and new HIV diagnoses through facility-based and community index testing

A total of 260,659 HIV tests were performed at the 102 HFs in the eight districts included from October 2017 to September 2018, with 10,673 new HIV diagnoses (4.1%) (Table 3). Worryingly, the number of clients tested declined from 473,947 in September 2019 to 306,987 by September 2020, and the number of new HIV diagnoses declined from 16,548 to 12,329 in the same period. In comparison, for community index testing, the total number of patients tested declined from 91,441 in September 2018 to 19,542 in September 2020 and the number of new HIV diagnoses declined from 7,011 to 2,728 in the same period; however, the percentage of new HIV diagnoses increased from 7.7% to 14.0%.

**Table 3:** Number of clients tested for HIV, number of new HIV diagnoses, and percentage of new HIV diagnoses through HF and community index testing.

Period	Gender	Clients tested				New HIV diagnoses				% new HIV diagnoses	
		# of clients tested		Percentage (%)		# of new HIV diagnoses		Percentage (%)			
		FT*	CIT**	FT	CIT	FT	CIT	FT	CIT	FT	CIT
October 2017-September 2018	Total	260,659	91,441	100	100	10,673	7,011	100	100	4.1	7.7
	Female	189,434	43,390	73	47	6,743	3,751	63	54	3.6	8.6
	Male	71,225	48,051	27	53	3,930	3,260	37	46	5.5	6.8
October 2018-September 2019	Total	473,947	46,190	100	100	16,548	3,655	100	100	3.5	7.9
	Female	354,102	24,157	75	52	10,767	1,970	65	54	3.0	8.2
	Male	119,845	22,033	25	48	5,781	1,685	35	46	4.8	7.6
October 2019-September 2020	Total	306,987	19,542	100	100	12,329	2,728	100	100	4.0	14.0
	Female	238,191	10,593	78	54	8,049	1,466	65	54	3.4	13.8
	Male	68,796	8,949	22	46	4,280	1,262	35	46	6.2	14.1

*FT—HF testing.

**CIT–Community index testing.

Overall, in the last three years, community index testing had higher percentage of new HIV diagnoses and a higher percentage of males screened and diagnosed with HIV. The percentage of male individuals tested through community index testing varied between 46%–53% whereas through facility-based testing this percentage varied between 22%-27%. A similar trend was observed for the number of new HIV diagnoses, 46% in community index testing vs 35–37% in facility-based testing, indicating that community index testing reaches proportionally more males.

## Discussion

This is the first study conducted in Mozambique that estimated the cost per client tested, new HIV diagnosis and per HIV infection averted for community index testing; it also highlighted that this testing strategy reaches proportionally more males than facility-based testing. From October 2017 to September 2020, males testing through community index testing was between 9–38% of all males tested and males identified as new HIV diagnoses in community index testing was 22–32% of all males identified as new HIV diagnoses, indicating a considerable contribution of community index testing (S4 Table in S1 File).

Facility-based index testing corresponded only to 0.2–1.2% of the total number of clients tested and 3.32–6.84% of the new HIV diagnosis (S4 Table in S1 File). Males tested through facility-based index testing corresponded only to 0.45–2.39% of all males tested and males identified as new HIV diagnosis through facility-based index testing corresponded to 5.38–9.95% of all males identified as new HIV diagnosis, indicating that only a small percentage of the people who need to be tested through index testing are actually tested in the HFs.

According to the latest UNAIDS report [1], globally, men continue to fare worse than women in terms of HIV testing, with one million more men than women living with an undiagnosed HIV infection. Our findings that community index testing reaches proportionally more males and has better percentage of new HIV diagnoses than facility-based testing, at a cost per new HIV diagnosis that is lower than published facility-based benchmarks in three countries in Southern Africa (Malawi, Zambia and Zimbabwe) (S3 Table in S1 File) [19], suggests that community index testing may be an effective and efficient strategy to increase identification of previously undiagnosed males.

The costs per client tested and per new HIV diagnosis in HFs in Malawi, Zambia and Zimbabwe were also sensitive to variations in the number of clients tested (and number of new HIV diagnoses), human resources, and costs of HIV rapid tests [19]. Because the cost per new HIV diagnosis—in both our study and Mwenge *et al*. [19]—is affected by the number of new HIV diagnoses, personnel, and costs of HIV test kits, the lower estimated cost reported here may be affected by differences in these parameters between locations.

A recent study in Zimbabwe by Vasantharoopan *et al*. [24], estimated that the cost per client tested via index-linked home-based HIV test using rapid tests delivered by healthcare worker, as done in our study, was $6.69 (in 2019 $) which is comparable to ours ($5.82).Our study does not include capital costs, but the pilot community index testing utilized minimal capital resources in its implementation, and therefore, capital or overhead costs are unlikely to have a substantial impact on these results. Thus, our estimated costs per client tested and per new HIV diagnosis would remain lower than those reported for facility-based [19] and comparable to index-linked home-based HIV testing [24], even if we considered capital costs.

An earlier study identified a strong relationship between cost per new HIV diagnosis and cost effectiveness for testing programs in low-income settings in southern Africa [25]. This strong relationship reported in Phillips *et al*. [25] further supports our finding that community index testing in Gaza is cost-effective. To our knowledge, there are no peer-reviewed published estimates of cost per infection averted per year for community index testing in sub-Saharan Africa. However, Okoboi *et al*. [21] estimated in Uganda that the cost-effectiveness of peer distributed HIV oral fluid self-test kits (a type of community HIV testing) in men who have sex with men and their social networks was $6,253 per infection averted per year whereas for standard-of-care hotspot testing was $17,567. Both of these estimates are much higher than $1,813 per infection averted per year estimated in the current study. The difference in the cost per infection averted between our study and Okoboi *et al*. [21] may be due to differences in the number of clients tested and new HIV diagnosis, salaries and price of HIV rapid tests which, as shown in this study, are major cost drivers. The generalizability of our cost per infection averted per year depends on the scale and efficiency of program implementation by the partners and the Ministry of Health.

This study identified the main cost drivers for community index testing in Mozambique, and the data generated here can be used to improve planning, budgeting, and resource allocation. Improved management of HIV testing is urgently needed, since external donor spending on HIV/AIDS in Africa has been declining significantly over the last few years; in 2015 alone, it declined by more than $1 billion [26,27]. African countries have been forced to increase their domestic budgets to fight the HIV/AIDS pandemic but face a wide range of constraints, including limited financial and human resources and debilitated infrastructure [26]. In addition, the COVID-19 pandemic caused major disruptions to healthcare systems, leading to supply shortages and diversion of human and financial resources [28].

Due to COVID-19 pandemic, there was a pronounced decrease in the overall number of people tested and new HIV diagnoses between October 2019 and September 2020. The pandemic forced temporary closure of facilities, staff shortages (due to contracting disease or undertaking COVID-19 related activities at the HF), and individuals being afraid to visit the HFs due to fear of exposure to the virus [29].

As the output and yield of facility-based testing decline (Table 3), and with the introduction of new testing modalities in Mozambique—most notably self-testing, which is in the early stages of rollout—it is important to understand the cost of resources required to implement testing strategies such as community index testing that complement facility-based testing. This analysis may help to increase that understanding.

## Conclusion

This study`s data suggests that the expansion of index testing would accelerate achieving the goal of identifying 95% of the people living with HIV by 2030 and would offer value for the investment. In addition, the current data suggest that community index testing may be an efficient strategy to increase identification of previously undiagnosed males. These findings show that analyses of program inputs are a useful tool to identify main cost drivers, inform planning and, improve efficiency and resource allocation in an era of declining funding.

## Dissemination

The evaluation report was shared with CDC and EGPAF global headquarters. A final evaluation report will be produced in alignment with PEPFAR Evaluation Standards of Practice requirements and posted on a publicly accessible website once approved by CDC.

The authors gave an oral presentation of the findings of this report at Mozambique’s XVII Jornadas Nacionais de Saúde, which took place in Maputo (Mozambique) from September 8–10, 2021. The findings were also presented in poster format at ICASA in Durban, South Africa, from December 6–11, 2021. Furthermore, the results of this study were presented and discussed with Gaza`s Provincial Health Directorate on March 17, 2022.

## Supporting information

S1 File (DOCX)
