# Supplementary material for: HIV community index testing reaches proportionally more males than facility-based testing and is cost-effective: A study from Gaza province, Mozambique
Source: PLoS One. 2023 May 26;18(5):e0286458. doi: 10.1371/journal.pone.0286458 (PMC10218717; doi:10.1371/journal.pone.0286458)
Supplement: S1 File — (DOCX) [file pone.0286458.s001.docx]

# Supporting information

**Table S1a.** Breakdown of total costs of testing and counseling**.**

| Cost Category | Total Cost | % of total cost |
| --- | --- | --- |
| Human resources | $299,245 | 52.40 |
| Supervisors | $25,664 | 4.49 |
| Data entry clerks and M&E | $18,480 | 3.24 |
| Field officers | $231,998 | 40.62 |
| Support staff | $10,508 | 1.84 |
| Total fringe | $12,595 | 2.21 |
| Travel and Transportation | $14,215 | 2.49 |
| Supervision | $10,324 | 1.81 |
| Transportation for field officers | $3,892 | 0.68 |
| Annualized training costs* | $34,820 | 6.10 |
| Testing and counseling training for field officers (Table S1b) | $41,349 |  |
| CommCare training for field officers (Table S1c) | $24,809 |  |
| Supplies | $43,108 | 7.55 |
| Gloves | $4,254 | 0.74 |
| Cotton wool (500 g) | $3,093 | 0.54 |
| Toilet paper | $937 | 0.16 |
| Glycerinated gel flask 80–100 ml | $3,239 | 0.57 |
| Cotton wool (500 g) | $1,979 | 0.35 |
| Face masks | $3,369 | 0.59 |
| Copies of invoices | $1,135 | 0.20 |
| Copies of family trees, diary of community health counseling and testing | $1,751 | 0.31 |
| Preprinted forms for monitoring and follow-up of all index cases | $812 | 0.14 |
| Register book for activists (100 pages each) | $1,053 | 0.18 |
| Depreciation in one year of smart phones purchased for field officers (Table S1d) | $21,485 | 3.76 |
| Communication and Review Meetings | $18,680 | 3.27 |
| Airtime and communication for field officers | $5,621 | 0.98 |
| Review meetings with field officers (district capital) | $10,813 | 1.89 |
| Review meetings with supervisors, M&E, and data entry staff | $2,246 | 0.39 |
| Subtotal—excluding HIV rapid tests | $410,068 | 71.80 |
| Screening for HIV with Determine HIV rapid test | $122,522 | 21.45 |
| Confirmation of HIV with Uni-Gold HIV rapid test | $38,522 | 6.75 |
| Subtotal—HIV testing | $161,044 | 28.20 |
| Total | **$571,112** | **100.00** |

*****Total training costs were annualized over 2 years (1), at a discount rate of 3% as recommended by WHO (2) and using an annuity of 1.9, similar to (3).

**Table S1b.** Cost of testing and counseling training for field officers.

| Category | Item | # | Unit cost (MZN) | Total cost (MZN) |
| --- | --- | --- | --- | --- |
| Duration | Number of days | 5 |  |  |
| Venue | Venue rent for 5 days | 1 | 5,000 | 25,000 |
| Lunch | Lunch for 5 days and 32 participants | 32 | 450 | 72,000 |
| Per diem | Per diem (400 MZN) for 30 field officers for 5 days | 30 | 400 | 60,000 |
|  | EGPAF facilitator (per diem + accommodations) | 1 | 6,350 | 31,750 |
|  | MOH facilitator (per diem + accommodations) | 1 | 6,000 | 30,000 |
|  | Subtotal—per diem | | | 218,750 |
| Total cost per training week | | | | 315,750 |
| Took 8 groups of 30 participants to train 250 field officers (250/30) | | 8 | 315,750 | 2,526,000 |
| Exchange rate (Oct. 2017) | | | | 61.09 |
| Total cost ($) | | | | **$41,349** |

**Table S1c.** Cost of commcare database training for field officers.

| Category | Item | # | Unit cost (MZN) | Total cost (MZN) |
| --- | --- | --- | --- | --- |
| Duration | Number of days | 3 |  |  |
| Venue | Venue rent for 3 days | 1 | 5,000 | 15,000 |
| Lunch | Lunch for 3 days and 32 participants | 32 | 450 | 43,200 |
| Per diem | Per diem (400 MZN) for 30 field officers for 3 days | 30 | 400 | 36,000 |
|  | EGPAF facilitator (per diem + accommodations) | 1 | 6,350 | 19,050 |
|  | MOH facilitator (per diem + accommodations) | 1 | 6,000 | 18,000 |
|  | Subtotal—per diem | | | 131,250 |
| Total cost per training week | | | | 189,450 |
| Took 8 groups of 30 participants to train 250 Field Officer (250/30) | | 8 | 189,450 | 1,515,600 |
| Exchange rate (Oct. 2017) | | | | 61.09 |
| Total cost ($) | | | | **$24,809** |

**Table S1d.** Cost of depreciation of smartphones purchased for field officers.

| Category | Value |
| --- | --- |
| Number of field officers | 250 |
| Cost per smart phone | MZN15,000 |
| Total cost | MZN3,750,000 |
| Exchange rate (Oct 2017) | MZN61.09 |
| Total cost ($) | **$61,384.84** |
| First year depreciation (35%) ($)^+^ | **$21,484.69** |

**^+^**According to Makov et al. (4), Samsung smartphones lose 35% of their value in the first year.

**Table S2a.** One-way sensitivity analysis of input categories (human resources, travel and transportation, supplies, and communication and review meetings) on cost per client tested and cost per new positive diagnosis.

|  | Category |  | Human resources | | Travel and Transportation | | Supplies | | Communication and Review Meetings | |
| --- | --- | --- | --- | --- | --- | --- | --- | --- | --- | --- |
|  |  | **Value** | **+10%** | **-10%** | **+10%** | **-10%** | **+10%** | **-10%** | **+10%** | **-10%** |
| Costs | Human resources | $299,245 | $329,169 | $269,320 | $299,245 | $299,245 | $299,245 | $299,245 | $299,245 | $299,245 |
|  | Travel and transportation | $14,215 | $14,215 | $14,215 | $15,637 | $12,794 | $14,215 | $14,215 | $14,215 | $14,215 |
|  | Supplies | $43,108 | $43,108 | $43,108 | $43,108 | $43,108 | $47,419 | $38,797 | $43,108 | $43,108 |
|  | Communication and review meetings | $18,680 | $18,680 | $18,680 | $18,680 | $18,680 | $18,680 | $18,680 | $20,548 | $16,812 |
|  | Annualized training costs | $34,820 | $34,820 | $34,820 | $34,820 | $34,820 | $34,820 | $34,820 | $34,820 | $34,820 |
|  | Subtotal—without HIV rapid tests | $410,068 | $439,993 | $380,144 | $411,490 | $408,647 | $414,379 | $405,757 | $411,936 | $408,200 |
|  | Screening—Determine HIV rapid test | $122,522 | $122,522 | $122,522 | $122,522 | $122,522 | $122,522 | $122,522 | $122,522 | $122,522 |
|  | Confirmation—Uni-Gold HIV rapid test | $38,522 | $38,522 | $38,522 | $38,522 | $38,522 | $38,522 | $38,522 | $38,522 | $38,522 |
|  | Subtotal—HIV rapid tests | $161,044 | $161,044 | $161,044 | $161,044 | $161,044 | $161,044 | $161,044 | $161,044 | $161,044 |
|  | Total | $571,112 | $601,036 | $541,187 | $572,533 | $569,690 | $575,423 | $566,801 | $572,980 | $569,244 |
| Number of clients | Clients tested | 91,441 | 91,441 | 91,441 | 91,441 | 91,441 | 91,441 | 91,441 | 91,441 | 91,441 |
|  | New HIV diagnoses | 7,011 | 7,011 | 7,011 | 7,011 | 7,011 | 7,011 | 7,011 | 7,011 | 7,011 |
| Cost per client excluding HIV rapid test | Cost per client tested | $4.48 | $4.81 | $4.16 | $4.50 | $4.47 | $4.53 | $4.44 | $4.50 | $4.46 |
|  | Cost per new HIV diagnosis | $58.49 | $62.76 | $54.22 | $58.69 | $58.29 | $59.10 | $57.87 | $58.76 | $58.22 |
| Price per HIV rapid test | Screening—Determine HIV rapid test | $1.34 | $1.34 | $1.34 | $1.34 | $1.34 | $1.34 | $1.34 | $1.34 | $1.34 |
|  | Confirmation—Uni-Gold HIV rapid test | $5.49 | $5.49 | $5.49 | $5.49 | $5.49 | $5.49 | $5.49 | $5.49 | $5.49 |
| Cost per subjects with HIV rapid test | Cost per client tested | $5.82 | $6.15 | $5.50 | $5.84 | $5.81 | $5.87 | $5.78 | $5.84 | $5.80 |
|  | Cost per new HIV diagnosis | $65.32 | $69.59 | $61.06 | $65.53 | $65.12 | $65.94 | $64.71 | $65.59 | $65.06 |
|  | **% Variation of cost/client tested** |  | **5.62** | **-5.62** | **0.27** | **-0.27** | **0.81** | **-0.81** | **0.35** | **-0.35** |
|  | **% Variation of cost/new HIV diagnosis** |  | **6.53** | **-6.53** | **0.31** | **-0.31** | **0.94** | **-0.94** | **0.41** | **-0.41** |

**Table S2b.** One-way sensitivity analysis of input categories (training, number of clients tested, number of clients tested positive, and price of rapid tests) on cost per client tested and cost per new positive diagnosis.

|  | Category |  | Training | | # clients tested | | # new HIV diagnoses | | Price of HIV rapid test | |
| --- | --- | --- | --- | --- | --- | --- | --- | --- | --- | --- |
|  |  | **Value** | **+10%** | **-10%** | **+10%** | **-10%** | **+10%** | **-10%** | **+10%** | **-10%** |
| Costs | Human resources | $299,245 | $299,245 | $299,245 | $299,245 | $299,245 | $299,245 | $299,245 | $299,245 | $299,245 |
|  | Travel and transportation | $14,215 | $14,215 | $14,215 | $14,215 | $14,215 | $14,215 | $14,215 | $14,215 | $14,215 |
|  | Supplies | $43,108 | $43,108 | $43,108 | $43,108 | $43,108 | $43,108 | $43,108 | $43,108 | $43,108 |
|  | Communication and review meetings | $18,680 | $18,680 | $18,680 | $18,680 | $18,680 | $18,680 | $18,680 | $18,680 | $18,680 |
|  | Annualized training costs | $34,820 | $38,302 | $31,338 | $34,820 | $34,820 | $34,820 | $34,820 | $34,820 | $34,820 |
|  | Subtotal—without HIV rapid tests | $410,068 | $413,550 | $406,586 | $410,068 | $410,068 | $410,068 | $410,068 | $410,068 | $410,068 |
|  | Screening—Determine HIV rapid test | $122,522 | $122,522 | $122,522 | $122,522 | $122,522 | $122,522 | $122,522 | $122,522 | $122,522 |
|  | Confirmation—Uni-Gold HIV rapid test | $38,522 | $38,522 | $38,522 | $38,522 | $38,522 | $38,522 | $38,522 | $38,522 | $38,522 |
|  | Subtotal—HIV rapid tests | $161,044 | $161,044 | $161,044 | $161,044 | $161,044 | $161,044 | $161,044 | $161,044 | $161,044 |
|  | Total | $571,112 | $574,594 | $567,630 | $571,112 | $571,112 | $571,112 | $571,112 | $571,112 | $571,112 |
| Number of clients | Clients tested | 91,441 | 91,441 | 91,441 | 100,585 | 82,297 | 91,441 | 91,441 | 91,441 | 91,441 |
|  | New HIV diagnoses | 7,011 | 7,011 | 7,011 | 7,011 | 7,011 | 7,712 | 6,310 | 7,011 | 7,011 |
| Cost per client excluding HIV rapid test | Cost per client tested | $4.48 | $4.52 | $4.45 | $4.08 | $4.98 | $4.48 | $4.48 | $4.48 | $4.48 |
|  | Cost per new HIV diagnosis | $58.49 | $58.99 | $57.99 | $58.49 | $58.49 | $53.17 | $64.99 | $58.49 | $58.49 |
| Price per HIV rapid test | Screening—Determine HIV rapid test | $1.34 | $1.34 | $1.34 | $1.34 | $1.34 | $1.34 | $1.34 | $1.47 | $1.21 |
|  | Confirmation—Uni-Gold HIV rapid test | $5.49 | $5.49 | $5.49 | $5.49 | $5.49 | $5.49 | $5.49 | $6.04 | $4.95 |
| Cost per subjects with HIV rapid test | Cost per client tested | $5.82 | $5.86 | $5.79 | $5.42 | $6.32 | $5.82 | $5.82 | $5.96 | $5.69 |
|  | Cost per new HIV diagnosis | $65.32 | $65.82 | $64.83 | $65.32 | $65.32 | $60.01 | $71.82 | $66.01 | $64.64 |
|  | **% Variation of cost/client tested** |  | **0.65** | **-0.65** | **-7.00** | **8.56** | **0.00** | **0.00** | **2.30** | **-2.30** |
|  | **% Variation of cost/new HIV diagnosis** |  | **0.76** | **-0.76** | **0.00** | **0.00** | **-8.14** | **9.95** | **1.05** | **-1.05** |

**Table S3.** Cost per client tested for HIV and cost per new HIV diagnosis at the health facilities in Malawi, Zambia and Zimbabwe.

| 2016 $ | | | | | | 2018 $ | | | | | |
| --- | --- | --- | --- | --- | --- | --- | --- | --- | --- | --- | --- |
| Cost per client tested | | | **Cost per new HIV diagnosis** | | | **Cost per client tested** | | | **Cost per new HIV diagnosis** | | |
| Malawi | Zambia | Zimbabwe | Malawi | Zambia | Zimbabwe | Malawi | Zambia | Zimbabwe | Malawi | Zambia | Zimbabwe |
| 4.92 | 4.24 | 8.79 | 79.58 | 73.63 | 178.92 | 5.15 | 4.44 | 9.20 | 83.26 | 77.04 | 187.19 |

**Source:** Mwenge *et al.* (5)for 2016 data. The costs were converted to 2018 using the US Consumer Price Index.

**Table S4.** Number of clients tested and new HIV diagnoses through community index and facility-based testing as percentage of total number of clients tested and new HIV diagnoses.

| Period |  | # of clients tested | | | | | | |  | # of new HIV diagnoses | | | | | | |
| --- | --- | --- | --- | --- | --- | --- | --- | --- | --- | --- | --- | --- | --- | --- | --- | --- |
|  | **Gender** | **Total** | **CIT** | **CIT as % of total** | **FT** | **FT as % of total** | **FIT*** | **FIT as % of total** |  | **Total** | **CIT** | **CIT as % of total** | **FT** | **FT as % of total** | **FIT** | **FIT as % of total** |
| October 2017-September 2018 | Total | 491,660 | 91,441 | 19 | 260,659 | 53 | ** | ** |  | 29,022 | 7,011 | 24 | 10,673 | 37 |  |  |
|  | Female | 365,776 | 43,390 | 12 | 189,434 | 52 |  |  |  | 18,868 | 3,751 | 20 | 6,743 | 36 |  |  |
|  | Male | 125,884 | 48,051 | 38 | 71,225 | 57 |  |  |  | 10,154 | 3,260 | 32 | 3,930 | 39 |  |  |
| October 2018-September 2019 | Total | 604,144 | 46,190 | 8 | 473,947 | 78 | 1,195 | 0.20 |  | 20,618 | 3,655 | 18 | 16,548 | 80 | 684 | 3.32 |
|  | Female | 435,482 | 24,157 | 6 | 354,102 | 81 | 443 | 0.10 |  | 13,102 | 1,970 | 15 | 10,767 | 82 | 280 | 2.14 |
|  | Male | 168,662 | 22,033 | 13 | 119,845 | 71 | 752 | 0.45 |  | 7,516 | 1,685 | 22 | 5,781 | 77 | 404 | 5.38 |
| October 2019-September 2020 | Total | 390,327 | 19,542 | 5 | 306,987 | 79 | 4,718 | 1.21 |  | 14,056 | 2,728 | 19 | 12,329 | 88 | 962 | 6.84 |
|  | Female | 292,950 | 10,593 | 4 | 238,191 | 81 | 2,388 | 0.82 |  | 9,049 | 1,466 | 16 | 8,049 | 89 | 464 | 5.13 |
|  | Male | 97,377 | 8,949 | 9 | 68,796 | 71 | 2,330 | 2.39 |  | 5,007 | 1,262 | 25 | 4,280 | 85 | 498 | 9.95 |

*FIT – facility-based index testing

** Information not available

**References**

1. Vyas S, Songo J, Guinness L, Dube A, Geis S, Kalua T, et al. Assessing the costs and efficiency of HIV testing and treatment services in rural Malawi: Implications for future “test and start” strategies. BMC Health Serv Res. 2020;20(1):740. Available from: https://bmchealthservres.biomedcentral.com/articles/10.1186/s12913-020-05446-5
2. WHO. WHO Guide to cost-effectiveness analysis. Geneva, Switzerland; 2003. Available from: https://www.who.int/choice/publications/p_2003_generalised_cea.pdf
3. Walker D, Kumaranayake L. Allowing for differential timing in cost analyses: discounting and annualization. Health Policy Plan. 2002;17(1):112–8. Available from: https://academic.oup.com/heapol/article/17/1/112/652244
4. Makov T, Fishman T, Chertow MR, Blass V. What Affects the Secondhand Value of Smartphones: Evidence from eBay. J Ind Ecol. 2019;23(3):549–59. Available from: https://onlinelibrary.wiley.com/doi/abs/10.1111/jiec.12806
5. Mwenge L, Sande L, Mangenah C, Ahmed N, Kanema S, D’Elbée M, et al. Costs of facility-based HIV testing in Malawi, Zambia and Zimbabwe. PLoS One. 2017 12(10). Available from: /pmc/articles/PMC5642898/
